# 16S rRNA-based genetic diversity and symbiotic efficiency of indigenous cowpea-nodulating rhizobia from semiarid Eastern Kenya

**DOI:** 10.3389/fmicb.2026.1875429

**Published:** 2026-06-10

**Authors:** Ahmed Kassem Khater, Charles Kibet Kirui, Ezekiel Mugendi Njeru, Stephen Mwangi Githiri

**Affiliations:** 1 Department of Molecular Biology and Biotechnology, Pan African University Institute for Basic Sciences, Technology and Innovation (PAUSTI), Nairobi, Kenya; 2 Department of Agricultural Biochemistry, Faculty of Agriculture, Al-Azhar University, Cairo, Egypt; 3 Department of Biochemistry, Microbiology and Biotechnology, Kenyatta University, Nairobi, Kenya; 4 Department of Horticulture and Food Security, Jomo Kenyatta University of Agriculture and Technology (JKUAT), Nairobi, Kenya

**Keywords:** 16S rRNA gene, biological nitrogen fixation, cowpea (*Vigna unguiculata*), rhizobia, semiarid Kenya, symbiotic efficiency

## Abstract

Indigenous rhizobia that nodulate cowpea (*Vigna unguiculata*) can support inoculant development for semiarid farming systems, yet their 16S rRNA-based molecular diversity and symbiotic performance in Eastern Kenya remain poorly characterized. In this study, we isolated nodule-associated bacteria from cowpea root nodules collected from smallholder farms in Machakos and Kitui Counties and evaluated nodulation and plant growth under greenhouse conditions. We recovered 70 isolates and grouped them into 19 morphotypes on the basis of colony and biochemical traits, but only 15 isolates (21.4%) formed nodules. The authenticated isolates varied in nodulation (16.20 ± 1.39 to 51.70 ± 4.68 nodules plant^−1^) and total dry biomass under greenhouse conditions. The symbiotic efficiency ranged from 37.88 to 157.04% relative to the nitrogen-supplemented control, and eight isolates (53.3%) exceeded 100%. Three isolates (M-34, M-17, and M-27) presented the highest efficiencies (~140–157%) and outperformed the nitrogen-supplemented control and the reference strain (*Bradyrhizobium* sp. USDA 3456). Partial 16S rRNA sequencing assigned isolates to *Rhizobium* (53.3%), *Bradyrhizobium* (40.0%), and one isolate was affiliated with *Mesorhizobium plurifarium*, representing one of the first documented associations of this species with cowpea in Eastern Africa. Closely related isolates differed markedly in efficiency, indicating that taxonomic identity alone does not predict symbiotic performance. These results identify high-performing indigenous strains for further evaluation as candidates for field evaluation and future inoculant development.

## Graphical abstract

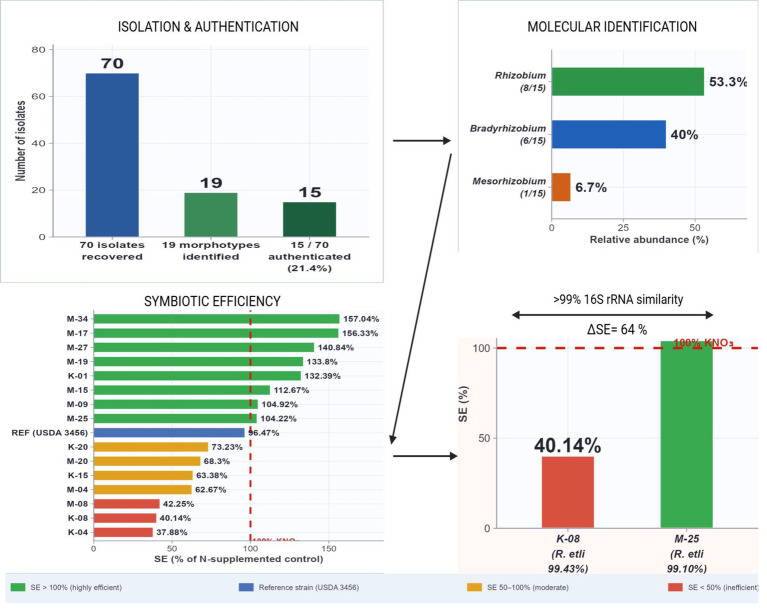

## 1 Introduction

Biological nitrogen fixation (BNF) is a natural process in which soil bacteria reduce atmospheric nitrogen (N₂) to ammonia, supplying plants with essential nutrients for growth (Barbieri et al., 2023). In legumes such as cowpea [*Vigna unguiculata* (L.) Walp.], BNF occurs through symbiosis with rhizobia that form root nodules, where nitrogen fixation takes place (Becker et al., 2024). Because BNF can reduce the plant dependence on synthetic nitrogen fertilizers and support sustainable soil fertility management, improving legume–rhizobia symbioses remain a key strategy for climate-resilient and low-input agriculture (Akchaya et al., 2025; Goyal et al., 2021).

Symbiotic effectiveness varies widely among rhizobial strains and depends on host–strain compatibility and environmental conditions, including soil pH, temperature, and water availability (Soumare et al., 2020; Goyal and Habtewold, 2023). In semiarid agroecosystems, low soil fertility and episodic water limitation commonly constrain crop productivity and can reduce nitrogen fixation benefits, making indigenous symbionts of local origin particularly important for region-specific inoculant development (Ben Gaied et al., 2024). Indigenous rhizobia may outperform introduced or nonlocal strains because of their familiarity with local soil conditions and potentially greater competitiveness for nodulation, though direct evidence of stress tolerance or ecological adaptation requires dedicated experimentation (Nyaga and Njeru, 2020). However, nodule-associated bacterial communities can be diverse, and their morphological resemblance to rhizobia does not necessarily indicate symbiotic competence or nitrogen-fixing capacity (Simbine et al., 2021). In addition, taxonomic identification based on conserved markers such as the 16S rRNA gene provides a useful phylogenetic context but does not consistently predict symbiotic performance, because key functional traits are influenced by symbiosis-related genes and their regulation (Antil et al., 2023).

Cowpea is a major grain legume in sub-Saharan Africa and is valued for its protein-rich seeds and tolerance to heat, drought, and poor soils (FAOSTAT, 2021). In Kenya’s arid and semiarid lands, cowpea contributes to food security and household income, yet yields often remain low due to nutrient limitations—particularly nitrogen—and variable responses to inoculation reported across environments and management systems (Amwata et al., 2016; Gerrano et al., 2019). Many studies in Africa report *Bradyrhizobium* as a frequent microsymbiont of cowpea (Ndungu et al., 2018), but recent molecular surveys indicate that the composition of cowpea-associated rhizobia can vary substantially across regions and agroecological conditions (Sena et al., 2020). This variability supports region-specific characterization of indigenous cowpea symbionts as a foundation for selecting effective strains for future inoculant development.

Furthermore, closely related rhizobial strains may differ markedly in their nitrogen-fixation performance, highlighting a potential mismatch between genetic relatedness and functional efficiency (Simbine et al., 2021; Epstein et al., 2023). In this context, we characterized indigenous cowpea-nodulating bacteria from smallholder farms in semiarid Machakos and Kitui Counties, Kenya, by isolating nodule-associated bacteria, confirming their nodulation ability under greenhouse conditions, quantifying symbiotic efficiency via plant biomass-based metrics, assigning taxonomic affiliation through 16S rRNA gene sequencing, and examining whether closely related isolates exhibit similar symbiotic performance. By combining functional screening with molecular identification, this study provides evidence-based insight into the diversity and effectiveness of indigenous cowpea symbionts in semiarid agroecosystems and identifies candidate strains for further evaluation as future inoculant candidates in semiarid cowpea systems.

## 2 Materials and methods

### 2.1 Study sites

**Figure 1:**
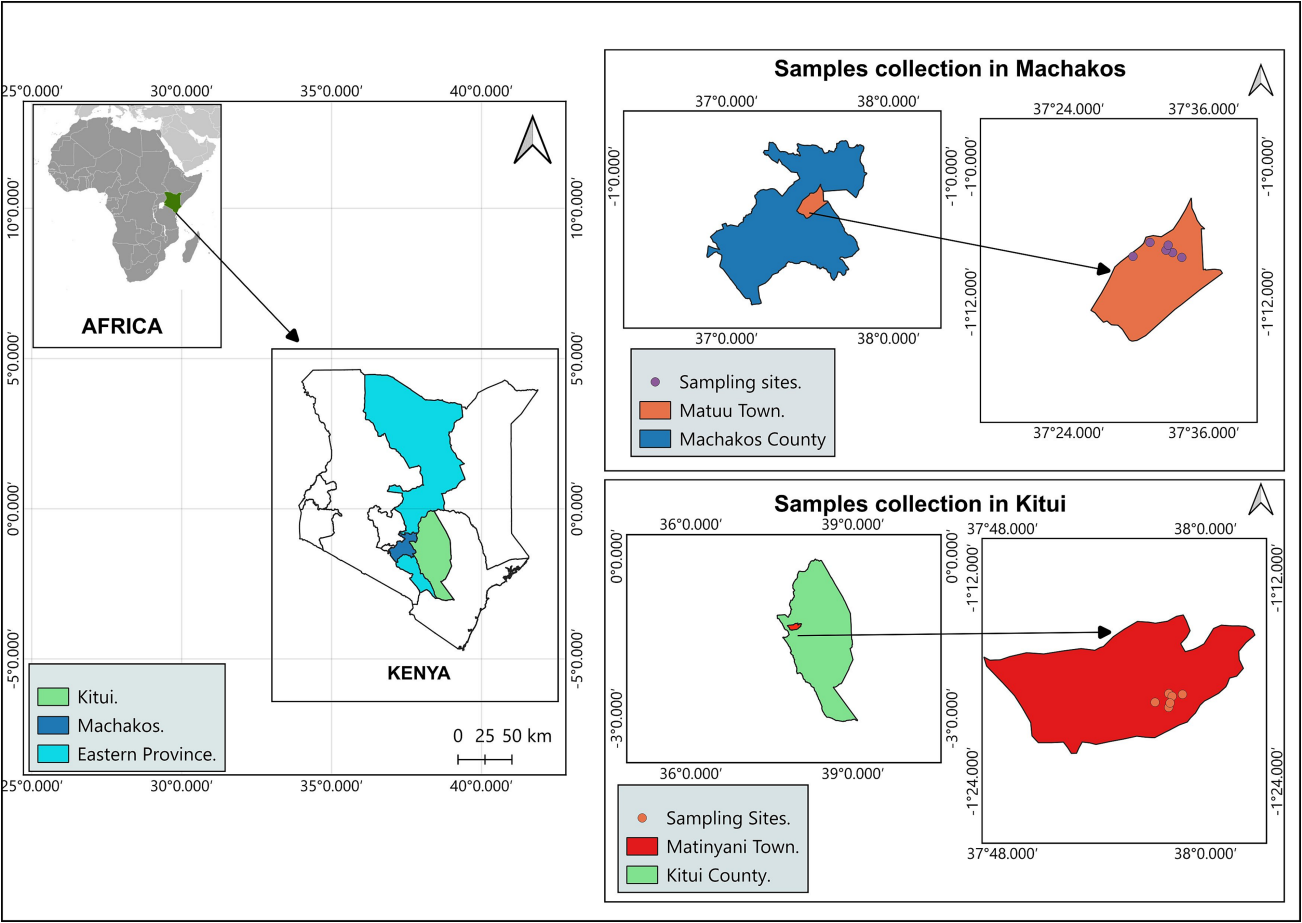
Geographic location of cowpea nodule sampling sites in semiarid Eastern Kenya. The left panel shows Kenya within the African continent, with the study region highlighted. Enlarged panels show the sampling areas within Machakos County (Matuu Town; 1°15′10”S, 37°54′28″E) and Kitui County (Matinyani Town; 1°19′03”S, 37°58′15″E). Dots indicate individual smallholder farm sampling sites (*n* = 6 per county). Maps were generated using QGIS.

The study was conducted on smallholder farms in semiarid Eastern Kenya. The farms were in Machakos County (near Matuu Town; 1°15′10”S, 37°54′28″E) and Kitui County (near Matinyani Town; 1°19′03”S, 37°58′15″E) (Figure 1). Twelve farms were selected (six per county). None had a history of commercial rhizobial inoculation. All the plants had been cultivated continuously for more than 3 years. The main crops grown were cowpea (*Vigna unguiculata*), green gram (*Vigna radiata*), common bean (*Phaseolus vulgaris*), pigeon pea (*Cajanus cajan*), and maize (*Zea mays*). The region is hot and dry. The mean annual temperature is 15–34 °C, and the annual rainfall is 639–881 mm on the basis of long-term climate records for the area (Source: Kenya Meteorological Department).

### 2.2 Root nodule collection and bacterial isolation

Nodules were collected from healthy cowpea plants at mid-flowering stage (45–50 days after planting). At each farm, 20 plants were sampled randomly. The roots were excavated (5–40 cm depth) via sterile hand shovels, and the nodules were removed with sterile forceps. The nodules were stored in sterile bottles with silica gel and transported to the Microbiology Laboratory at Kenyatta University, Nairobi, on ice. The samples were processed within 48 h (Barillot et al., 2013).

The nodules were surface-sterilized (Somasegaran and Hoben, 2012) and rehydrated in sterile distilled water for 2 h. They were immersed in 70% ethanol for 30 s, then treated with 3% sodium hypochlorite for 3 min and rinsed six times with sterile distilled water. The sterilized nodules were crushed aseptically in sterile distilled water. A loopful of the suspension was streaked on yeast extract mannitol agar (YEMA) supplemented with Congo red (0.025 g/L). Congo red was incorporated into the medium because true rhizobia characteristically do not absorb the dye and appear white to cream on the medium, whereas contaminating non-rhizobial bacteria typically produce pink or red colonies, thereby serving as a selective visual indicator during primary isolation (Somasegaran and Hoben, 2012). The plates were incubated at 28 °C in darkness and monitored for up to 7 days. Typical colonies that did not absorb Congo red in the dark were subcultured on fresh YEMA–Congo red plates. Purification was repeated four times, and the pure isolates were stored in 20% glycerol–YEM broth at −80 °C (Muindi et al., 2021).

### 2.3 Morphological and biochemical characterization

The colony morphology was recorded after 7 days on YEMA at 28 °C. The traits included colony size, shape, margin, color, opacity, texture, and elevation (Brenner et al., 2015). Gram staining was performed on 3-day-old cultures, which were subsequently examined at 1000 × magnification. The bromothymol blue (BTB) reaction was tested on YEMA containing bromothymol blue (0.025 g L^−1^; pH 6.8). The plates were incubated at 28 °C for 7 days. Yellow coloration indicates acid production (fast growers), whereas blue coloration indicates alkali production (slow growers). Congo red absorption was also assessed on YEMA–Congo red. On the basis of morphological and biochemical traits consistent with those of rhizobia, 70 isolates were selected for nodulation authentication (Somasegaran and Hoben, 2012).

### 2.4 Authentication of cowpea-nodulating rhizobia

Nodulation was tested in a greenhouse via modified Leonard jars (Somasegaran and Hoben, 2012; Muthini, 2014). The conditions used were natural light (~12 h photoperiod), 21–28 °C, and 60–78% relative humidity. Leonard jars were prepared via plastic cups (8 cm top diameter) with cotton wicks connected to 1-L vessels containing sterile nitrogen-free nutrient solution. The plastic cups and 1-L vessels were sterilized by soaking in 70% ethanol for 30 min, followed by UV irradiation under a laminar flow hood for 60 min, whereas cotton wicks were autoclaved at 121 °C for 20 min.

The vermiculite was soaked overnight, washed for 2 days with frequent water changes, and sterilized. Cowpea seeds [K-80; sourced from Kenya Agricultural and Livestock Research Organization (KALRO)] were surface sterilized in 3% sodium hypochlorite for 5 min and then rinsed six times with sterile distilled water. Three seeds were planted per jar. The seedlings were thinned to two plants after 5 days (Muthini, 2014).

Each isolate was grown in YEM broth to the midexponential phase (OD₆₀₀ = 0.6–0.8; ~10^8^ CFU mL^−1^). At day 8 after planting, the seedlings were inoculated with 1 mL of culture. Two controls were included: (i) a positive control (uninoculated supplied with 0.05% KNO₃) and (ii) a negative control (uninoculated supplied with only nitrogen-free nutrient solution). The design was a randomized complete block design with five replicates per treatment.

Fifty days after inoculation, plants were harvested and evaluated for nodulation and biomass accumulation. Isolates that induced formation of at least one nodule were classified as nodule-forming under the greenhouse conditions applied (Somasegaran and Hoben, 2012). From the tested isolates, those that induced nodule formation under greenhouse conditions were selected for further symbiotic efficiency testing and molecular characterization.

### 2.5 Symbiotic efficiency determination

Symbiotic efficiency was evaluated via the experimental setup described in Section 2.4. In addition to the controls, a reference strain (*Bradyrhizobium* sp. strain USDA 3456) was included for comparison. At harvest, the shoots and the roots were separated, and the fresh weights were recorded. Nodules were detached and counted. The plant tissues were dried at 65 °C to a constant weight. The shoot dry weight (SDW), root dry weight (RDW), and total dry weight (TDW = SDW + RDW) were calculated.

The symbiotic efficiency (SE) was calculated as follows:

$$\mathrm{SE}\;(\%)=(\frac{\mathrm{TDW}\;\text{of inoculated plants}}{\mathrm{TDW}\;\text{of nitrogensupplemented control}})\times 100$$

The nitrogen-supplemented control was defined as 100% efficiency. Isolates were classified as highly efficient (SE > 100%), moderately efficient (50–100%), or inefficient (SE < 50%). It should be noted that SE, as calculated from plant biomass, serves as a proxy for overall symbiotic benefit under controlled greenhouse conditions and does not directly measure nitrogen fixation activity. Direct quantification of nitrogen fixation would require additional parameters such as acetylene reduction assays (ARA), total plant nitrogen analysis, nodule dry weight, or leghemoglobin content.

### 2.6 Molecular characterization

#### 2.6.1 Genomic DNA extraction

DNA was extracted from 2-day-old pure cultures via the Quick-DNA Fungal/Bacterial Miniprep Kit (D-6005; Zymo Research, USA). DNA was quantified via a NanoDrop spectrophotometer (Jenway Genova Nano, UK), whereas the DNA quality thresholds were A₂₆₀/A₂₈₀ = 1.8–2.0 and A₂₆₀/A₂₃₀ > 1.8. DNA integrity was checked on a 1.0% agarose gel stained with GelRed in 0.5 × TBE buffer.

#### 2.6.2 16S rRNA gene amplification and sequencing

The 16S rRNA gene was amplified via the universal bacterial primers 27F (5’-AGAGTTTGATCCTGGCTCAG-3′) and 1492R (5’-GGTTACCTTGTTACGACTT-3′) (Weisburg et al., 1991). The PCR mixture (25 μL total volume) contained: 12.5 μL of OneTaq 2 × Master Mix with Standard Buffer (New England Biolabs, Ipswich, MA, USA), 0.5 μL of each primer (10 μM), 1.0 μL of template DNA (50–100 ng/μL), and 10.5 μL of nuclease-free water.

The thermal cycling conditions were as follows: initial denaturation at 95 °C for 5 min; 30 cycles of denaturation at 95 °C for 45 s, annealing at 58 °C for 30 s, and extension at 72 °C for 90 s; and a final extension at 72 °C for 7 min. PCR products were stored at 4 °C prior to gel analysis and sequencing. Negative controls without template DNA were included in each amplification run to monitor contamination. The PCR products were checked on a 1.4% agarose gel. Amplicons (~1.5 kb) were sequenced bidirectionally via Sanger sequencing (Macrogen Europe B. V., Amsterdam, Netherlands).

#### 2.6.3 Sequence analysis and phylogenetic reconstruction

Chromatograms were inspected in FinchTV v1.4.0. Low-quality bases (Phred <20) were trimmed (Treves, 2010). The reads were assembled into consensus sequences in BioEdit v7.2.5 (Hall, 1999). The sequences were identified via BLASTn against NCBI GenBank. Reference sequences of type strains showing ≥97% 16S rRNA gene sequence similarity to the query isolates were retrieved from NCBI GenBank and included in the phylogenetic analysis. The alignments were generated via ClustalW in MEGA X v10.2. The best-fit nucleotide substitution model was selected using the Bayesian Information Criterion (BIC) in MEGA X v10.2, which identified the Tamura 3-parameter model with Gamma-distributed rates (T92 + G) as optimal. The phylogenetic tree was reconstructed using the Maximum Likelihood (ML) method with the T92 + G substitution model, 1,000 bootstrap replicates, and the Nearest-Neighbor-Interchange (NNI) heuristic search. Bootstrap support values ≥70% are shown at branch nodes (Kumar et al., 2018). *Paraburkholderia phenoliruptrix* strain AC1100 (NR_042901.1) was used as the outgroup.

The sequences were deposited in GenBank under the following accession numbers: PX765075, PX765076, PX765077, PX765078, PX765079, PX765080, PX765081, PX765082, PX765083, PX765084, PX765086, PX765087, PX765088, PX765090, and PX765091.

### 2.7 Statistical analysis

Morphological diversity indices (Shannon H′, Simpson 1-D, dominance, and equitability) were calculated via PAST v3.0 as descriptive metrics of phenotypic heterogeneity within the isolate pool. These indices were solely to characterize variation among colony morphotypes prior to nodulation screening. Morphotypes were grouped via UPGMA clustering with Gower’s similarity coefficient as a preliminary visual summary of phenotypic groupings to guide isolate selection before authentication.

Greenhouse data (nodule number, SDW, RDW, TDW, and SE) were analyzed via one-way ANOVA. Mean separation was performed via Tukey’s HSD test (*α* = 0.05). The results are reported as means ± SEM. Statistical significance was set at *p* < 0.05 unless stated otherwise.

### 2.8 Biosafety considerations

All microbiological work followed standard aseptic procedures. Personal protective equipment was used when handling the bacterial cultures and DNA-staining reagents. Waste was disposed of according to institutional biosafety guidelines.

## 3 Results

### 3.1 Morphological and biochemical characterization of indigenous isolates

We recovered 70 bacterial isolates from cowpea nodules collected from 12 smallholder farms in Machakos and Kitui Counties, Eastern Kenya. Hierarchical cluster analysis of morphological and biochemical traits grouped the isolates into 19 morphotypes (G-01 to G-19; Table 1).

**Table 1:** Morphological and biochemical characteristics of 19 colony morphotypes derived from 70 nodule-associated bacteria recovered from cowpea (*Vigna unguiculata*) roots in semiarid Eastern Kenya.

Morphotype	Colony shape	Margin	Color	Colony size	Opacity	Texture	Elevation	GRAM STAIN	BTB reaction	Congo red absorption	No. of isolates
G-01	Irregular	Irregular	White	Medium	Opaque	Dry	Flat	−ve	Blue	Cna	4
G-02	Irregular	Irregular	White	Small	Opaque	Dry	Flat	−ve	Blue	Cna	5
G-03	Circular	Smooth	Creamy White	Small	Opaque	Dry	Flat	−ve	Blue	Cna	3
G-04	Circular	Smooth	White	Small	Opaque	Dry	Flat	−ve	Blue	Cna	4
G-05	Circular	Smooth	White	Medium	Opaque	Dry	Flat	−ve	Blue	Cna	4
G-06	Circular	Smooth	White	Small	Opaque	Dry	Flat	−ve	Blue	Cna	4
G-07	Irregular	Smooth	Watery White	Medium	Translucent	Mucoid	Convex	−ve	Yellow	Cna	2
G-08	Circular	Irregular	Watery White	Medium	T-O	Mucoid	Convex	−ve	Yellow	Cna	2
G-09	Circular	Smooth	Watery White	Medium	T-O	Mucoid	Raised	−ve	Yellow	Cna	2
G-10	Circular	Smooth	Watery White	Medium	T-O	Mucoid	Convex	−ve	Yellow	Cna	7
G-11	Circular	Smooth	White	Medium	T-O	Mucoid	Convex	−ve	Yellow	Cna	4
G-12	Circular	Smooth	Watery White	Large	T-O	Mucoid	Convex	−ve	Yellow	Cna	4
G-13	Irregular	Smooth	Watery White	Large	T-O	Mucoid	Convex	−ve	Yellow	Cna	4
G-14	Circular	Smooth	Watery White	Large	Translucent	Mucoid	Convex	−ve	Yellow	Cna	3
G-15	Circular	Smooth	White	Small	Opaque	Mucoid	Raised	−ve	Yellow	Cna	3
G-16	Circular	Smooth	White	Medium	Opaque	Mucoid	Raised	−ve	Yellow	Cna	5
G-17	Circular	Smooth	White	Small	Opaque	Mucoid	Convex	−ve	Yellow	Cna	1
G-18	Circular	Smooth	White	Large	Opaque	Mucoid	Convex	−ve	Yellow	Cna	2
G-19	Circular	Smooth	White	Medium	Opaque	Mucoid	Convex	−ve	Yellow	Cna	7

T-O, translucent with opaque center; −ve, gram-negative; Cna, Congo red nonabsorber. The colony size categories were small (<2 mm), medium (2–4 mm), and large (>4 mm).

**Figure 2:**
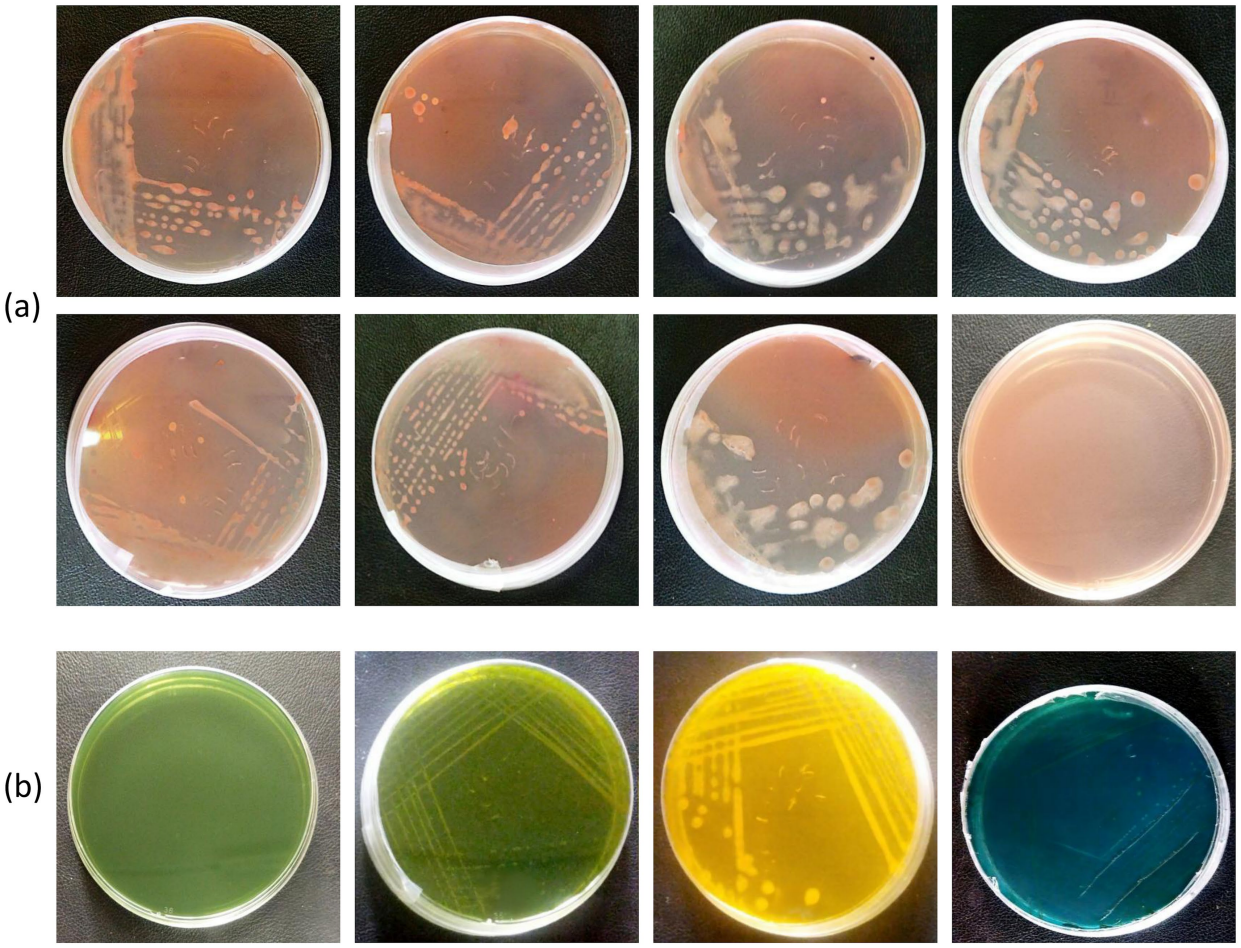
Morphological and biochemical characterization of nodule-associated bacterial isolates. **(a)** Representative colony morphotypes on yeast extract mannitol agar (YEMA) supplemented with Congo red (0.025 g L^−1^) after 7 days of incubation at 28 °C, showing variation in colony size, shape, elevation, and texture. **(b)** Growth reactions on YEMA supplemented with bromothymol blue (BTB; 0.025 g L^−1^; pH 6.8) showing the spectrum from strong acid production (yellow; fast-growing isolates) to alkali production (blue; slow-growing isolates) after 7 days at 28 °C.

The isolates presented clear phenotypic variation in YEMA after 7 days at 28 °C (Figure 2a). The colony size ranged from small (<2 mm) to large (>4 mm), with medium colonies (2–4 mm) predominating. Most morphotypes (78.9%) formed circular colonies, whereas four morphotypes presented irregular forms. Smooth margins predominated (84.2%), and most colonies appeared white. The colonies were either opaque (57.9%) or translucent with opaque centers (six morphotypes). Most isolates produced mucoid colonies (68.4%), and colony elevation was mainly convex (52.6%). All 70 isolates presented poor Congo red absorption and appeared white to cream on YEMA–Congo red.

The results of biochemical tests supported the rhizobial profile (Table 1). All the isolates were gram-negative. The BTB test separated the isolates according to their pH response (Figure 2b). Acid production (yellow; pH < 6.0) occurred in 68.4% of the morphotypes (13/19), representing 46 isolates. Alkali production (blue; pH > 7.6) occurred in 31.6% of the morphotypes (6/19), representing 24 isolates.

Descriptive diversity metrics indicated substantial phenotypic heterogeneity in the colony morphotype pool in both counties (Table 2). The Shannon index (H′) was 2.797 in Machakos and 2.597 in Kitui, and the Simpson index (1-D) was 0.9395 and 0.9193, respectively. Dominance was low (0.0605 in Machakos; 0.0806 in Kitui), and equitability was high (0.9315 in Machakos; 0.9136 in Kitui). Machakos yielded 16 morphotypes from 35 isolates, and Kitui yielded 14 morphotypes from 35 isolates.

**Table 2:** Descriptive phenotypic diversity indices of colony morphotypes derived from nodule-associated bacteria recovered from cowpea (*Vigna unguiculata*) in semiarid Machakos and Kitui Counties, Eastern Kenya.

Index	Kitui	Machakos
Taxa_S	14	16
Individuals	35	35
Dominance_D	0.0806	0.0605
Simpson_1-D	0.9193	0.9395
Shannon_H	2.597	2.797
Evenness_eH/S	0.9587	1.0250
Brillouin	1.9640	2.0810
Equitability_J	0.9136	0.9315

**Figure 3:**
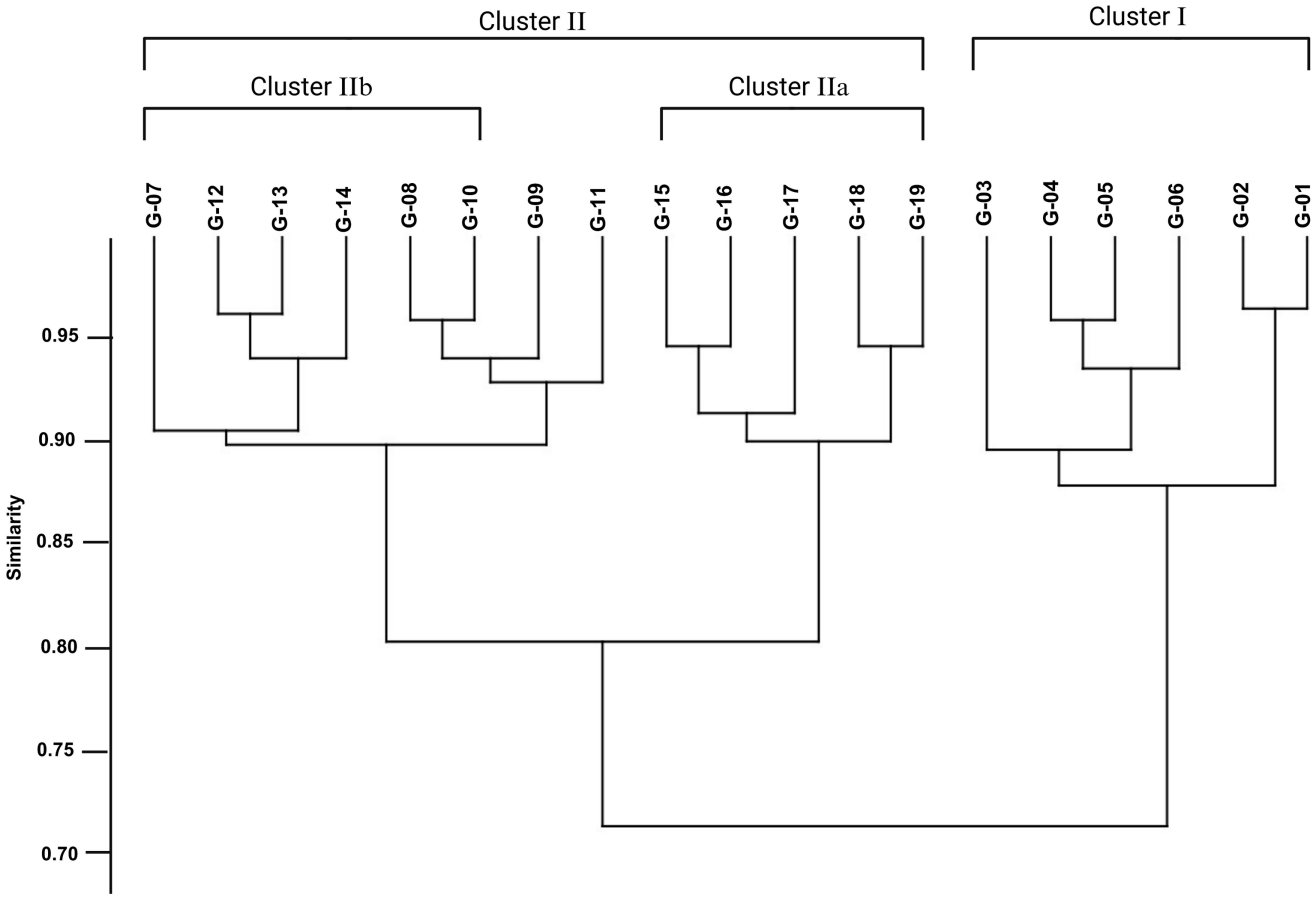
Phenotypic similarity-based clustering of 19 colony morphotypes derived from nodule-associated bacteria recovered from cowpea in semiarid Eastern Kenya. The dendrogram was constructed using the unweighted pair group method with arithmetic mean (UPGMA) algorithm based on Gower’s similarity coefficient calculated from morphological and biochemical traits in PAST v3.0. Two major phenotypic clusters are resolved at 0.70 similarity: Cluster I comprises six morphotypes producing alkaline reactions on YEMA–BTB with dry colony texture, and Cluster II comprises 13 morphotypes producing acid reactions with mucoid colony texture, further subdivided into subclusters IIa and IIb at approximately 0.80 similarity. The similarity scale is indicated on the *y*-axis.

UPGMA clustering via Gower’s similarity coefficient resolved two major clusters at 0.70 similarity (Figure 3). Cluster I contained six morphotypes (G-01 to G-06). These morphotypes mainly produced alkaline reactions on BTB, with a dry colony texture and flat elevation. Cluster II contained 13 morphotypes (G-07 to G-19). These morphotypes produced acid reactions on BTB and resulted in mucoid colonies. Cluster II further separated into two subclusters (IIa and IIb) at ~0.80 similarity. Across the dataset, BTB reaction, colony texture, and elevation contributed most to phenotypic separation.

### 3.2 Authentication of cowpea-nodulating rhizobia and evaluation of symbiotic efficiency

In greenhouse assays, 15 of 70 isolates (21.4%) induced nodule formation on cowpea roots within 50 days after inoculation (Table 3). The remaining 55 isolates (78.6%) did not induce nodule formation under the test conditions. The nitrogen-supplemented positive control (PC) and the uninoculated negative control (NC) remained nonnodulated throughout the experiment.

**Table 3:** Nodulation, plant growth, and symbiotic efficiency of authenticated cowpea-nodulating rhizobial isolates under greenhouse conditions.

Isolate ID	Morphotype	County	Nodules plant^−1^	Shoot dry weight (g plant^−1^)	Root dry weight (g plant^−1^)	Symbiotic efficiency (%)
K-01	G-03	Kitui	49.20 ± 3.70^a^	1.50 ± 0.05^ab^	0.38 ± 0.04^abcd^	132.39 ± 14.33^ab^
K-04	G-13	Kitui	18.00 ± 1.51^c^	0.42 ± 0.04^fg^	0.11 ± 0.03^e^	37.88 ± 2.63^e^
K-08	G-08	Kitui	16.20 ± 1.39^c^	0.45 ± 0.02^fg^	0.12 ± 0.03^e^	40.14 ± 1.45^e^
K-15	G-19	Kitui	20.80 ± 2.26^c^	0.74 ± 0.13^efg^	0.16 ± 0.02^e^	63.38 ± 5.93^de^
K-20	G-10	Kitui	20.20 ± 1.82^c^	0.83 ± 0.03^def^	0.21 ± 0.03^bcde^	73.23 ± 6.41^cde^
M-04	G-08	Machakos	17.40 ± 1.80^c^	0.72 ± 0.05^efg^	0.17 ± 0.04^de^	62.67 ± 6.65^de^
M-08	G-13	Machakos	23.40 ± 3.37^c^	0.47 ± 0.02^fg^	0.13 ± 0.04^e^	42.25 ± 2.66^e^
M-09	G-10	Machakos	37.10 ± 0.88^b^	1.19 ± 0.08^cde^	0.30 ± 0.02^abcde^	104.92 ± 5.52^bcd^
M-15	G-02	Machakos	37.40 ± 3.01^b^	1.28 ± 0.10^bc^	0.32 ± 0.03^abcde^	112.67 ± 12.32^abc^
M-17	G-01	Machakos	51.70 ± 4.68^a^	1.75 ± 0.09^ab^	0.47 ± 0.06^a^	156.33 ± 5.72^a^
M-19	G-02	Machakos	35.40 ± 2.50^b^	1.51 ± 0.04^abc^	0.39 ± 0.04^abc^	133.80 ± 12.11^ab^
M-20	G-18	Machakos	23.40 ± 0.97^c^	0.77 ± 0.03^def^	0.20 ± 0.02^cde^	68.30 ± 4.72 ^cde^
M-25	G-11	Machakos	36.00 ± 1.94^b^	1.18 ± 0.16^cde^	0.30 ± 0.03^abcde^	104.22 ± 16.19^bcd^
M-27	G-03	Machakos	43.60 ± 3.24^ab^	1.58 ± 0.14^abc^	0.42 ± 0.02^ab^	140.84 ± 19.07^ab^
M-34	G-02	Machakos	44.60 ± 0.76^ab^	1.80 ± 0.14^a^	0.43 ± 0.02^a^	157.04 ± 18.26^a^
PC	–	–	00.00 ± 00^d^	1.13 ± 0.08^cde^	0.29 ± 0.06^abcde^	100 ± 0.00^bcd^
REF	–	–	15.40 ± 1.02^c^	1.20 ± 0.03^cde^	0.17 ± 0.03^de^	96.47 ± 5.79^bcd^
NC	–	–	00.00 ± 00^d^	0.23 ± 0.03^g^	0.12 ± 0.02^e^	24.64 ± 2.10^e^
*p*-value	–	–	P < 0.0001	*p* < 0.0001	P < 0.0001	P < 0.0001

Values are means ± SEM (n = 5). Different lowercase letters within each column indicate significant differences among treatments (Tukey’s HSD test, *p* < 0.05). PC, uninoculated positive control supplied with 0.05% KNO₃; REF, reference inoculant (*Bradyrhizobium* sp. strain USDA 3456); NC, uninoculated negative control supplied with nitrogen-free nutrient solution. TDW, total dry weight. SE = (TDW of inoculated plants / TDW of nitrogen-supplemented control) × 100.

Authenticated isolates varied widely in nodulation. The nodule number ranged from 16.20 ± 1.39 to 51.70 ± 4.68 plant^−1^, and the treatments differed significantly (*p* < 0.0001). Isolate M-17 produced the most nodules (51.70 ± 4.68), followed by K-01 (49.20 ± 3.70), M-34 (44.60 ± 0.76), and M-27 (43.60 ± 3.24). These isolates showed statistically comparable nodulation. These values represented ~2.8-fold to 3.3-fold greater nodulation than did the reference strain (15.40 ± 1.02). Isolates M-09, M-15, M-19, and M-25 formed 35.40–37.40 nodule plant^−1^ (~2.3-fold to 2.4-fold higher than the reference strain). Overall, 13 of the 15 authenticated isolates (86.6%) produced significantly more nodules than did the reference strain, with increases ranging from 16.9 to 235.7%.

Ten authenticated isolates (66.7%) originated from Machakos County and five (33.3%) originated from Kitui County. The authenticated isolates represented 10 morphotypes (G-01, G-02, G-03, G-08, G-10, G-11, G-12, G-13, G-18, and G-19). Acid-producing morphotypes accounted for 70% of the authenticated isolates, whereas alkali-producing morphotypes accounted for 30%. Plant growth variables differed significantly among treatments (*p* < 0.0001; Table 3). Isolate M-34 produced the highest shoot dry weight and total biomass, while K-04 showed the lowest values across all growth parameters.

Isolate M-34 produced the highest shoot dry weight (1.80 ± 0.14 g) and exceeded all the other treatments, including the nitrogen-supplemented control. In terms of root dry weight, M-17 (0.47 ± 0.06 g) and M-34 (0.43 ± 0.02 g) presented the highest dry weight, followed by M-27 (0.42 ± 0.02 g). For total biomass, M-34 (2.23 g) and M-17 (2.22 g) had the highest values. These values exceeded those of the nitrogen-supplemented control (1.42 g) by 57.0 and 56.3%, respectively, and exceeded those of the reference strain (1.37 g) by 62.8 and 62.0%, respectively. M-27 ranked third (2.00 g), exceeding the nitrogen-supplemented control and reference strain by 40.8 and 46.0%, respectively. K-01 (1.88 g), M-19 (1.90 g), and M-15 (1.60 g) also exceeded those of both controls.

Symbiotic efficiency (SE) ranged from 37.88 to 157.04% among authenticated isolates. Eight isolates (53.3%) were highly efficient (SE > 100%). These isolates were M-34 (157.04 ± 18.26%), M-17 (156.33 ± 5.72%), M-27 (140.84 ± 19.07%), M-19 (133.80 ± 12.11%), K-01 (132.39 ± 14.33%), M-15 (112.67 ± 12.32%), M-09 (104.92 ± 5.52%), and M-25 (104.22 ± 16.19%). The reference strain (*Bradyrhizobium* sp. USDA 3456) achieved a symbiotic efficiency of 96.47 ± 5.79%. The top five isolates (M-34, M-17, M-27, M-19, and K-01) had SE values >130%. Four isolates showed moderate efficiency (62–73%): K-20 (73.23 ± 6.41%), M-20 (68.30 ± 4.72%), K-15 (63.38 ± 5.93%), and M-04 (62.67 ± 6.65%). Three isolates were inefficient (SE < 50%) despite nodulation, M-08 (42.25 ± 2.66%), K-08 (40.14 ± 1.45%), and K-04 (37.88 ± 2.63%).

### 3.3 16S rRNA-based taxonomic characterization and phylogenetic placement

**Figure 4:**
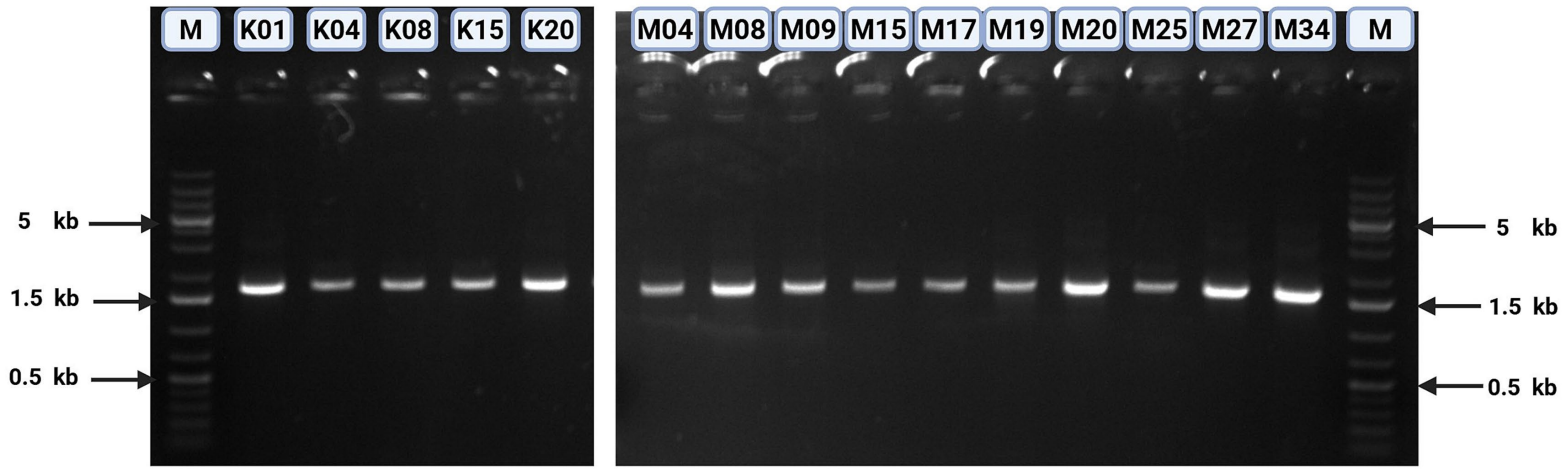
Agarose gel electrophoresis of 16S rRNA gene PCR amplicons from 15 authenticated cowpea-nodulating isolates. PCR was performed using universal bacterial primers 27F and 1492R. A single amplicon of approximately 1.5 kb was obtained from all isolates, confirming successful and specific amplification of the 16S rRNA gene. Lanes labeled M indicate the 1 kb DNA molecular weight marker. Lane labels correspond to individual isolate IDs (K-01 to K-20 and M-04 to M-34). The 1.5 kb and 0.5 kb marker bands are indicated by arrows.

PCR amplification of the 16S rRNA gene from all 15 authenticated isolates produced single amplicons of ~1.5 kb (Figure 4). All amplicons were suitable for bidirectional sequencing. BLASTn analysis revealed 96.94–99.65% similarity to reference type strains (Table 4). The query coverage ranged from 97 to 100%, and all the E-values were 0.0. The 15 isolates were affiliated with three genera: *Rhizobium* (53.3%; 8/15), *Bradyrhizobium* (40.0%; 6/15), and *Mesorhizobium* (6.7%; 1/15).

**Table 4:** 16S rRNA gene sequence similarity of 15 authenticated cowpea-nodulating isolates to closest type strain matches in the NCBI GenBank database.

Isolate ID.	GenBank accession number	Top BLAST match	Similarity (%)	Query coverage (%)	E-value
K-01	PX765075	*Bradyrhizobium elkanii*	99.65	98	0.0
K-04	PX765076	*Rhizobium alamii*	99.50	100	0.0
K-08	PX765077	*Rhizobium etli*	99.43	98	0.0
K-15	PX765079	*Rhizobium mesosinicum*	99.19	99	0.0
K-20	PX765078	*Rhizobium leguminosarum*	99.16	99	0.0
M-04	PX765080	*Mesorhizobium plurifarium*	98.87	99	0.0
M-08	PX765081	*Rhizobium leguminosarum*	98.97	100	0.0
M-09	PX765083	*Rhizobium tropici*	99.43	99	0.0
M-15	PX765084	*Bradyrhizobium* sp.	99.58	99	0.0
M-17	PX765082	*Bradyrhizobium japonicum*	99.58	99	0.0
M-19	PX765086	*Bradyrhizobium elkanii*	99.40	99	0.0
M-20	PX765088	*Rhizobium tropici*	99.46	99	0.0
M-25	PX765087	*Rhizobium etli*	99.23	98	0.0
M-27	PX765091	*Bradyrhizobium yuanmingense*	99.44	98	0.0
M-34	PX765090	*Bradyrhizobium japonicum*	99.65	99	0.0

Similarity values represent pairwise sequence identity (%) to the closest type strain match. Query coverage (%) and E-values were obtained from BLASTn searches against the NCBI GenBank nucleotide database. Taxonomic affiliations represent 16S rRNA-based preliminary genus-level placements and should not be regarded as confirmed species identifications.

Within *Bradyrhizobium*, the isolates presented the closest similarity to *B. elkanii* (K-01: 99.65%; M-19: 99.40%), *B. japonicum* (M-17: 99.58%; M-34: 99.65%), *Bradyrhizobium* sp. (M-15: 96.94%), and *B. yuanmingense* (M-27: 99.44%). The *Rhizobium* isolates matched *R. alamii* (K-04: 99.50%), *R. etli* (K-08: 99.43%; M-25: 99.10%), *R. mesosinicum* (K-15: 99.19%), *R. leguminosarum* (K-20: 99.16%; M-08: 98.95%), and *R. tropici* (M-09: 99.43%; M-20: 99.46%). The *Mesorhizobium* isolate (M-04) showed 98.87% 16S rRNA gene similarity to *M. plurifarium*, representing a tentative affiliation pending higher-resolution molecular confirmation. We deposited all the sequences in GenBank (PX765075–PX765091).

**Figure 5:**
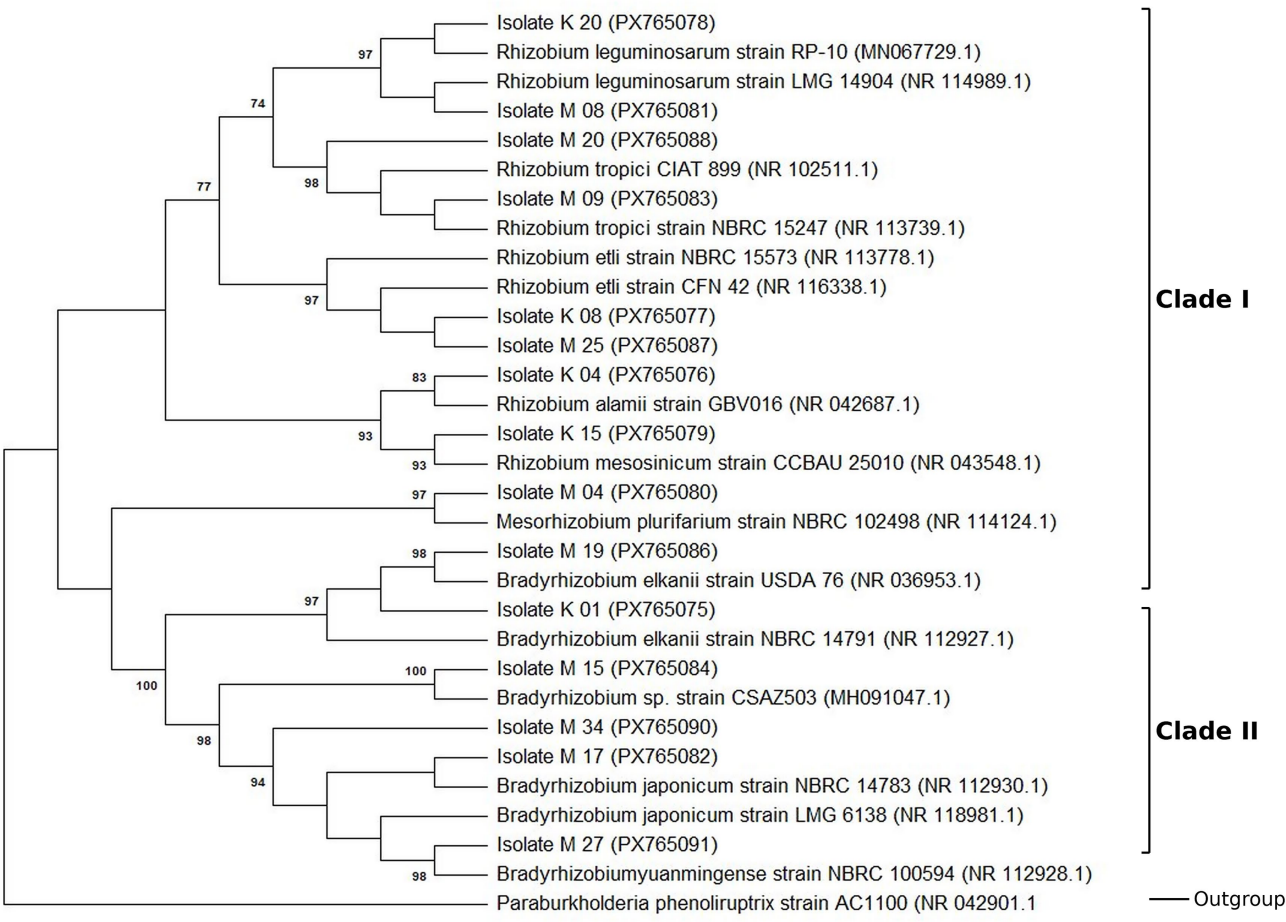
Maximum likelihood phylogenetic tree of 15 authenticated cowpea-nodulating isolates based on partial 16S rRNA gene sequences (~1.5 kb). Bootstrap support values ≥70% are shown at branch nodes. Isolates from this study are indicated in bold with GenBank accession numbers in parentheses. The tree resolves two main clades: clade I comprising isolates with 16S rRNA-based affiliation to *Rhizobium* and *Mesorhizobium*, and clade II comprising isolates affiliated with *Bradyrhizobium*. *Paraburkholderia phenoliruptrix* strain AC1100 (NR_042901.1) was used as the outgroup.

Maximum Likelihood phylogenetic analysis (T92 + G model; 1,000 bootstrap replicates) resolved two main clades (Figure 5). The tree included *Paraburkholderia phenoliruptrix* strain AC1100 (NR_042901.1) as the outgroup. Clade I contained the *Rhizobium* and *Mesorhizobium* affiliates with strong internal bootstrap support (74–97%). This clade comprised subclusters containing the *R. leguminosarum*-affiliated isolates K-20 and M-08 (97%), the *R. tropici*-affiliated isolates M-09 and M-20 (98%), the *R. etli*-affiliated isolates K-08 and M-25 (97%), the *R. alamii*-affiliated isolate K-04 (83%), the *R. mesosinicum*-affiliated isolate K-15 (93%), and the *M. plurifarium*-affiliated isolate M-04 forming a distinct lineage (97%). Clade II contained all *Bradyrhizobium* affiliates with strong bootstrap support (100% at the clade root). Within Clade II, M-19 and K-01 grouped with *B. elkanii* reference strains (97–98%), M-15 grouped with *Bradyrhizobium* sp. CSAZ503 (100%), M-34 and M-17 grouped with *B. japonicum* reference strains (94%), and M-27 grouped with *B. yuanmingense* (98%).

A particularly notable finding was the lack of concordance between 16S rRNA similarity and symbiotic performance. Despite sharing >99% 16S rRNA sequence similarity and affiliation with *R. etli*, isolates K-08 and M-25 differed markedly in symbiotic efficiency (40.14% vs. 104.22% SE respectively). Similarly, K-20 and M-08, both affiliated with *R. leguminosarum*, showed contrasting performance (73.23% vs. 42.25% SE). K-01 and M-19 are both affiliated with *B. elkanii* (>99% similarity) and presented similar SE values (132.39 and 133.80%) but differed in nodule number (49.20 vs. 35.40 nodules plant^−1^). M-17 and M-34 were both affiliated with *B. japonicum* (>99% similarity) and presented similar SE (156.33 and 157.04%), but M-17 produced more nodules than did M-34 (51.70 vs. 44.60 nodules plant^−1^).

## 4 Discussion

This study evaluated the 16S rRNA-based molecular diversity and symbiotic performance of indigenous cowpea nodule isolates from semiarid Eastern Kenya, with the aim of identifying functionally effective strains for future inoculant development. We recovered 70 bacterial isolates from cowpea nodules sampled across Machakos and Kitui Counties. However, only 15 isolates (21.4%) induced nodule formation under greenhouse conditions. This outcome demonstrates that colony morphology and routine biochemical screening alone cannot reliably identify nodule-competent cowpea microsymbionts. Several studies have reported that legumes host diverse nodule-associated endophytes, many of which do not establish functional nitrogen-fixing symbioses (Leite et al., 2017; De Meyer et al., 2015; Etesami, 2022; Ngwenya et al., 2022). Our results therefore reinforce the importance of greenhouse nodulation assays, especially when the goal is to identify candidates for inoculant development or symbiotic efficiency evaluation (Díaz-Rodríguez et al., 2025).

Phenotypic screening grouped the isolates into 19 morphotypes, indicating substantial heterogeneity in the nodule-associated bacterial pool in these semiarid agroecosystems. Acid-producing morphotypes predominated (68.4%), whereas alkali-producing morphotypes constituted 31.6%. While many reports describe cowpea as being commonly nodulated by slow-growing *Bradyrhizobium* (Pule-Meulenberg et al., 2010), the predominance of acid-producing groups in our collection is consistent with patterns reported from other semiarid and water-limited environments, where fast-growing rhizobia have been associated with competitive advantages under nutrient-limited conditions (Lagunas et al., 2023; Li et al., 2012). Whether this pattern reflects environmental selection under the specific soil conditions of Machakos and Kitui Counties cannot be determined without soil physicochemical data, and this interpretation should be regarded as speculative. The descriptive phenotypic metrics showed moderate to high variation in colony morphotypes across both counties (H′ = 2.597–2.797); however, the low authentication rate (21.4%) confirms that phenotypic heterogeneity does not necessarily translate into functional symbiotic diversity. This outcome reinforces the importance of complementing morphology-based grouping with nodulation authentication and biomass-based performance evaluation (Wekesa et al., 2021). Among the authenticated isolates, several strains demonstrated strong greenhouse growth promotion responses. Compared with the nitrogen-supplemented control, isolates M-34, M-17, and M-27 showed high symbiotic performance (~140–157% SE) and outperformed the reference strain (*Bradyrhizobium* sp. USDA 3456) in terms of total plant biomass production. Studies in Kenya and other regions frequently report that indigenous isolates can match or surpass commercial strains under local conditions (Nyaga and Njeru, 2020; Mathu et al., 2012; Mulas et al., 2011). Kyei-Boahen et al. (2023) reported moderate symbiotic efficiency for commercial inoculants. Together, these comparisons support the view that indigenous strains of local origin can provide strong symbiotic performance under greenhouse conditions, although field validation remains essential. Whether this performance advantage reflects specific adaptation to local soil or climatic conditions was not directly tested in the present study and would require dedicated soil characterization and stress tolerance assays.

We also observed that number of nodules alone did not consistently predict symbiotic efficiency. For example, M-17 produced the most nodules (51.70 nodules plant^−1^) and achieved very high efficiency (156.33% SE), but K-01 produced a comparable number of nodules (49.20 nodules plant^−1^) but achieved a lower efficiency (132.39% SE). Conversely, M-19 produced fewer nodules (35.40 nodules plant^−1^) but achieved 133.80% SE. These patterns indicate that nodule quantity alone does not fully predict symbiotic performance or plant growth benefit. Nodule effectiveness likely varies with nodule size, internal physiology, bacteroid activity, and host regulatory factors, which collectively influence the net growth benefit to the plant under greenhouse conditions (Girija et al., 2020; Huisman and Geurts, 2019). Therefore, selecting elite inoculant strains requires biomass- and efficiency-based screening rather than relying on nodule counts alone.

16S rRNA sequencing assigned the authenticated isolates to *Rhizobium* (53.3%), *Bradyrhizobium* (40.0%), and *Mesorhizobium* (6.7%). Cowpea is recognized as a relatively promiscuous host and can form nodules with diverse alphaproteobacterial genera (Puozaa et al., 2019). The presence of both fast-growing (*Rhizobium*) and slow-growing (*Bradyrhizobium*) taxa among authentic isolates suggests that cowpea in semiarid Eastern Kenya can associate with multiple lineages. The detection of a *Mesorhizobium plurifarium*-like isolate nodulating cowpea is notable. While *Mesorhizobium* species have been reported as legume symbionts in other regions (Wang et al., 2003), documentation of such associations in Eastern Africa appears limited. Because 16S rRNA gene sequencing provides only preliminary genus-level taxonomic placement and lacks sufficient resolution for definitive species delineation in rhizobia, the taxonomic assignments reported here should be regarded as 16S rRNA-based affiliations rather than confirmed species identifications. Definitive species-level confirmation and robust evolutionary interpretation would require higher-resolution approaches, including multilocus sequence analysis (MLSA) targeting housekeeping genes such as *recA, atpD, and glnII*, symbiotic gene characterization (*nodA, nifH*), or whole-genome sequencing. Future studies incorporating these approaches would strengthen the taxonomic conclusions and clarify phylogeographic patterns among cowpea-nodulating rhizobia in Eastern Africa.

A key finding was the lack of a simple relationship between 16S rRNA similarity and symbiotic performance. Isolates with highly similar 16S rRNA sequences presented large differences in symbiotic efficiency. For example, K-08 and M-25 are both affiliated with *R. etli* (>99% similarity), yet their efficiencies differ substantially (40.14% vs. 104.22% SE). This genotype–phenotype interaction has been reported across many legume–rhizobia interactions and reflects the fact that symbiotic function depends heavily on nodulation and nitrogen fixation genes and their regulation, which can vary independently of conserved ribosomal genes (Andrews et al., 2018). Thus, 16S rRNA-based identification provides a useful taxonomic context but cannot substitute for functional screening when inoculant candidates are selected (de Souza et al., 2025).

Overall, the elite isolates identified in this study—particularly M-34, M-17, and M-27, which exceeded the nitrogen-supplemented control by 40–57% in total biomass—represent promising candidate strains for future field evaluation. However, it must be emphasized that greenhouse performance under controlled conditions does not necessarily translate into field competitiveness, persistence, or inoculant success under natural agricultural conditions, due to competition with resident soil rhizobia, variability in soil physicochemical properties, and genotype-by-environment interactions (Vaidya and Stinchcombe, 2020). Multilocation field trials assessing nodule occupancy, performance stability across seasons, and compatibility across cowpea varieties are therefore essential before any practical inoculant recommendation can be made (Thuita et al., 2012; Kawaka et al., 2014). In addition, optimization of inoculant formulation and carrier materials, together with integration into broader soil fertility management practices, will be necessary to maximize adoption and impact (Bashan et al., 2014; Koskey et al., 2018). It should also be acknowledged that symbiotic efficiency in this study was assessed using total plant dry biomass as a proxy metric, following the approach of Muindi et al. (2021) and Nyaga and Njeru (2020). Direct nitrogen fixation was not measured; future studies should incorporate acetylene reduction assays, total plant nitrogen analysis, nodule dry weight, and leghemoglobin content to more rigorously quantify the nitrogen fixation contribution of these isolates.

## 5 Conclusion

This study revealed that cowpea nodules in semiarid Eastern Kenya host phenotypically and molecularly diverse nodule-associated bacteria, but only a subset of these bacteria form nodule-forming associations with plant growth promotion. Among the 70 isolates, 15 (21.4%) formed nodules and promoted plant growth under greenhouse conditions, confirming that morphology-based selection alone does not reliably identify functional rhizobia. On the basis of 16S rRNA sequences, authenticated isolates affiliated with *Rhizobium* (53.3%), *Bradyrhizobium* (40.0%), and *Mesorhizobium* (6.7%), including a *Mesorhizobium plurifarium*-like isolate, were identified. Three isolates (M-34, M-17, and M-27) presented the highest symbiotic efficiency (~140–157%) and outperformed the nitrogen-supplemented control and the reference strain. Finally, isolates with highly similar 16S rRNA sequences differed markedly in symbiotic efficiency, indicating that taxonomic identity alone does not predict performance. These results identify promising indigenous strains as candidates for field evaluation and future inoculant development and support validation through field trials and competitiveness testing.

## Data Availability

The 16S rRNA gene sequences generated in this study have been deposited in the NCBI GenBank database under accession numbers PX765075–PX765091. All other data supporting the findings of this study are available from the corresponding author upon reasonable request.
