# Correction: 16S rRNA-based genetic diversity and symbiotic efficiency of indigenous cowpea-nodulating rhizobia from semiarid Eastern Kenya

**DOI:** 10.3389/fmicb.2026.1913845

**Published:** 2026-07-08

**Authors:** Frontiers Production Office

**Affiliations:** Frontiers Media SA, Lausanne, Switzerland

**Keywords:** 16S rRNA gene, biological nitrogen fixation, cowpea (*Vigna unguiculata*), rhizobia, semiarid Kenya, symbiotic efficiency

Affiliation Max-Planck-Institute for Chemical Ecology, Germany was omitted for reviewer Clabe Wekesa. Friedrich Schiller University Jena, Germany was removed.

Max-Planck-Institute for Chemical Ecology, Germany has now been added for the reviewer Clabe Wekesa.

The original version of this article has been updated.

